# The Relationships Between Vitamin K and Cognition: A Review of Current Evidence

**DOI:** 10.3389/fneur.2019.00239

**Published:** 2019-03-19

**Authors:** Ludovico Alisi, Roberta Cao, Cristina De Angelis, Arturo Cafolla, Francesca Caramia, Gaia Cartocci, Aloisa Librando, Marco Fiorelli

**Affiliations:** ^1^Department of Sense Organs, Sapienza University of Rome, Rome, Italy; ^2^Department of Radiology, IRCCS San Raffaele Scientific Institute, Milan, Italy; ^3^Department of Radiological, Oncological and Anatomo-Pathological Sciences, Sapienza University of Rome, Rome, Italy; ^4^Department of Cell Biotechnology and Hematology, Sapienza University of Rome, Rome, Italy; ^5^Department of Human Neuroscience, Sapienza University of Rome, Rome, Italy

**Keywords:** vitamin K, phylloquinone, cognitive impairment, vitamin K antagonists, warfarin

## Abstract

Vitamin K is a fat-soluble nutrient discovered in 1935 and its role in blood coagulation has been thoroughly explored. In recent years, studies conducted *in vitro* and on animals highlighted vitamin K involvement in brain cells development and survival. In particular, vitamin K seems to have an antiapoptotic and anti-inflammatory effect mediated by the activation of Growth Arrest Specific Gene 6 and Protein S. Moreover, this vitamin is involved in sphingolipids metabolism, a class of lipids that participate in the proliferation, differentiation, and survival of brain cells. An altered expression in sphingolipids profile has been related to neuroinflammation and neurodegeneration. This review stems from a growing interest in the role of vitamin K in brain functions, especially in cognition, also in view of an expected increase of prevalence of Alzheimer's disease and other forms of dementia. It collects recent researches that show interesting, even though not definitive, evidence of a direct correlation between vitamin K levels and cognitive performance. Moreover, vitamin K antagonists, used worldwide as oral anticoagulants, according to recent studies may have a negative influence on cognitive domains such as visual memory, verbal fluency and brain volume. The aim of this review is to analyze the evidence of clinical studies carried out up to date on the relationship between vitamin K intake and cognitive performances. The involvement of vitamin K antagonists (VKAs) in declining cognitive performances is also addressed separately.

## Introduction

Vitamin K is a fat-soluble nutrient mainly found in green leafy vegetables as phylloquinone (Vitamin K1). This vitamin is widely known for its procoagulant effect. It acts as a cofactor for the enzyme that allows the activation of vitamin K-dependent factors (II, VII, IX, X, protein C, and protein S). A recent review collected studies that show its involvement in the metabolism of the central nervous system (CNS), suggesting the possibility that a vitamin K deficiency might be related to the onset of cognitive impairment (1).

These recently discovered functions, revealed that this vitamer participate in the enzymatic activation of growth-arrest specific 6 protein (Gas-6) and protein S. The first has an anti-apoptotic, mitogenic, and myelinating activity, the latter offers neuronal protection during ischemic/hypoxic injury both *in vivo* and *in vitro* (2–4). Furthermore, vitamin K is known to be an inductor of sphingolipids synthesis. These polar lipids are an essential part of CNS cell membrane and are linked to neuronal proliferation and differentiation (1). Several studies are also investigating the correlation between cognitive impairment and the use of vitamin K antagonists (VKAs, i.e., warfarin, acenocoumarol, and fluindion) as oral anticoagulants. In fact, these Coumarin derivatives prevent the recycle of vitamin K after the ɤ-carboxylation (5).

Due to the progressive aging of global population, data indicate that cases of dementia will double between 2020 and 2040, rising up to 81 million and consequently burdening society and national health systems. For instance, Alzheimer's disease (AD) cases in the US will rise from 5.4 million up to 13.8 million by mid-century (6, 7). Hence the importance of identifying modifiable factors that could impact the course of neurodegenerative pathologies. The aim of this review is to analyze the evidence of clinical studies carried out up to date examining the hypothesis of a cognitive decline among adults with low serum levels or dietary intake of vitamin K. Moreover, studies evaluating the potential link between VKAs and cognitive functions were also included.

## Dementia and Cognitive Impairment

Dementia can be defined as a clinical syndrome of mental capacity characterized by a substantial global decline in cognitive function that is not attributable to altered consciousness; it consists of a combination of symptoms attributable to various causes or pathological events (8). Cognitive impairment is a definition used in this review to indicate alterations in multiple cognitive domains highlightable with standardized tests, as clinically manifest dementia is often preceded by a heterogeneous spectrum of cognitive performances (9).

It is difficult to find univocal data about the prevalence of cognitive impairment and other forms of dementia (10); some studies show that the global prevalence of dementia varies among different countries, this could be related to a large number of variables including education, mean age, socioeconomic level, lack of a comparable methodology (11). However, it can certainly be affirmed that the two most common type of dementia in Western countries are Alzheimer's disease (up to 60% of cases) and vascular dementia (up to 20% of cases). These two forms of dementia are easily mistaken one for another due to their similarities in symptomatology, pathophysiology, and risk factors (12).

The mechanism underlying Alzheimer's disease is the deposition of β-amyloid peptide (Aβ) and the neurofibrillary tangles of the microtubule binding protein tau. In particular, Aβ peptides are responsible for the massive neuronal death that defines the disease (13).

A few studies concluded that Vitamin K seems to prevent Aβ-induced apoptosis through the activation of Gas-6, showing a pro-survival effect on brain cells (14).

Regarding vascular dementia, the main causes are represented by several vascular pathologies that result in cerebral ischemia. Studies published in the last years have attributed to Protein S (activated by vitamin K) a role in improving post-ischemic cerebral blood flow (15) and potentially leading to a more favorable cognitive outcome.

## Vitamin K Structure and Function

Vitamin K can be found as phylloquinone (the main dietary source of vitamin K) and it's also identified as menaquinones (vitamin K2) which include several vitamers of bacterial origin (2). Menaquinone-4 (MK-4) is the most represented vitamer in both human and rats' brains (16, 17).

MK-4 seems to protect against oxidative damage and inflammatory cascade activation in *in vitro* studies (18, 19). In addition, in murine models MK-4 depletion has been found correlated with worse cognitive performances (20).

Vitamin K is widely known for its role in blood coagulation as the cofactor of ɤ-glutamyl carboxylase that allows the activation of vitamin K-dependent factors such as factor II, VII, IX, X, protein C, and protein S. Vitamin K is also involved in the ɤ-carboxylation of two vitamin K-dependent proteins whose activity contributes to an adequate cerebral homeostasis, namely Gas-6 and protein S (3, 4). Moreover, vitamin K participates as a cofactor in the synthesis of sphingolipids, an important constituent of brain cells membrane (21). Several studies conducted on *in vitro* and murine models have highlighted the role of these constituents in brain metabolism. In some cases, a correlation with neurodegenerative diseases emerged that could be further examined trough human studies.

### Gas-6

Gas-6 has a central role in the development and survival of nervous system. In addition, it shows an anti-apoptotic, mitogenic, and myelinating activity in neuronal and glial cells (1).

Gas-6 binds and activates the receptor tyrosine kinases of the Tyro3, Axl, and Mer (TAM) family. Axl is involved in the proliferation of numerous cell types and in the survival of gonadotropin-releasing hormone (GnRH) neurons allowing their migration from the olfactory bulb to the hypothalamus (22, 23).

Mer protects primary macrophages from oxidative stress induced-apoptosis (24).

The specific role of Tyro3 in cell survival is yet to be defined, but activities similar to Axl have been observed concerning the migration of GnRH neurons A (25–27).

An *in vitro* study revealed that recombinant Gas-6 protects hippocampal rats' neurons from apoptosis, underlining the pro-survival effect of this protein through the activation of TAM proteins (28).

Through the activation of Axl and phosphatidylinositol 3-kinase (PI3K) pathways, Gas-6 modulates oligodendrocyte survival and microglial phenotype both *in vitro* and *in vivo* (3) preventing tumor necrosis factor alpha-induced apoptosis. A study on cuprizone-induced demyelination model (adopted as a model of multiple sclerosis) revealed that the deletion of Gas-6/Axl signaling leads to prolonged neuroinflammation with axonal damage and consistent demyelination (29, 30). This immune-regulatory role links Gas-6 to autoimmune disorders, more specifically to the pathogenesis of multiple sclerosis (MS) (31, 32).

Studies developed using murine culture of microglial cells showed that Gas-6 downregulates the expression of Interleukin-1b e induced nitric oxide synthase, thereby reducing the proinflammatory response (33). Two recent murine models using knock-out mice for Mer and Axl, demonstrated a reduced recruitment of microglial cells to neuronal sites of injury, also affecting the phagocytic activity through cytoskeleton changes (34, 35).

Furthermore, Gas-6 has shown a decrease of ß-amyloid-induced apoptosis, a hallmark of Alzheimer's disease, through the inhibition of low-voltage Ca^2+^ influx channels (14). However, a more recent study found that Gas-6 inhibits Tyro3 whose effects prevent β-amyloid deposition (36).

### Protein S

Historically protein S has been known for its anticoagulant effect, but recent studies are exploring further effects, such as a possible role in inflammation, angiogenesis, and cancer (37). As well as in ameliorating post-ischemic cerebral blood flow (15).

Protein S shares with Gas-6 almost half of its amino acid structure (44%) (4), and consequently it performs part of its actions as a TAM receptor ligand. Zhu et al. showed a direct correlation between the inhibition of Tyro3/Akt signaling pathway and the hypoxic-induced apoptosis of hippocampal neurons, underlining a potential protective effect of protein S in cerebral infarct (38). In particular, protein S has a protective effect against NMDA-induced toxicity and apoptosis via the Tyro3/Akt pathway (39). This finding may suggest a possible role of protein S as an adjunct of tissue Plasminogen Activator to reduce cerebral post-ischemic toxicity (40), without increasing the risk of bleeding when administered alone in high concentration to stroke rodent models (15). An additional property of protein S seems to be the preservation of the integrity of the blood-brain barrier (BBB) as it operates as a safeguard against chronic ischemic damage and BBB-related disorders (41).

### Sphingolipid Metabolism

Sphingolipids is one of the major classes of eukaryotic lipids and an essential component of cell membranes and their synthesis is known to be induced by vitamin K (21).

The sphingolipids most frequently observed in neuronal cell membranes are ceramides, sphingomyelin, cerebrosides, sulfatides, and gangliosides (42). This class of lipids seems to be a vital modulator of cell proliferation, differentiation, and survival (43). A growing amount of evidence are associating sphingolipids metabolism to the pathophysiology of CNS diseases. These polar lipids have been related to a neuroinflammatory and neurodegenerative states due to microglial activation and accumulation of amyloid precursor protein (APP) (44).

These are at the basis of the development of several pathologies like AD (43, 45), where inflammation is a consequence of microglial activation triggered by β-amyloid plaques (46).

Lastly, sphingolipids guide the process of myelination in the CNS and are themselves major components of oligodendrocyte membrane. In the serum and cerebrospinal fluid of patients affected by multiple sclerosis antibodies have been detected against myelinic sphingolipids (47, 48) along with ceramide accumulation in active lesions (49).

### The Assessment of Vitamin K Status

Both biomarkers and questionnaires have been suggested to evaluate vitamin K status. Circulating phylloquinone can be measured with high performance liquid chromatography (HPLC) a method that responds to changes in dietary phylloquinone intake. Serum phylloquinone should be measured in fasting samples to better reflect overall nutritional status. Moreover, vitamin K serum levels are influenced by serum triglycerides and should be corrected accordingly. There is currently no established threshold of circulating phylloquinone that indicates insufficiency or deficiency (50).

Other circulating markers are: PIVKA-II (protein induced by vitamin K absence-II) that has shown low sensitivity to dietary variation of vitamin K; undercarboxylated fraction of osteocalcin (unOc) and desphospho-uncarboxylated matrix Gla-protein (dp-ucMGP) that are both more sensitive than PIVKA-II but still conditioned by factors such as age and the total amount of matrix Gla-proteins available (50).

Vitamin K intake can be assessed with questionnaires such as the food frequency questionnaire (FFQ) or dietary records. While being efficient in terms of costs and time and easy to implement, questionnaires rely on the recall ability and perceptions of individuals' diet and therefore may be subjected to bias (51).

Considering the lack of a single gold standard measure, and the limitations affecting the available methods, vitamin K status may be best assessed with a combination of both questionnaires and biomarkers (50).

## VKAs-Brain Relationship

VKAs exercise their function by blocking the activity of vitamin K oxidoreductase (VKOR) preventing the recycle of vitamin K after the ɤ-carboxylation (5). The possibility of an adverse impact of VKAs on the brain has been evident since the finding of abnormalities of the CNS in newborns exposed to warfarin or other coumarin derivatives (52). However, detailed mechanisms are yet to be comprehended. Studies on whether Gas-6 and protein S ɤ-carboxylation are impaired by VKAs in the human brain have not yet been conducted. Studies performed on murine models have shown how VKAs determine a decrease in MK-4 brain concentrations, the most represented vitamer in rats' brain (16). Nagakawa et al. identificated the enzyme responsible for the conversion of phylloquinone in MK-4 (UBIAD1) (17) and Tamadon-Nejad et al. demonstrated that despite an excess of phylloquinone in rats' brain, MK-4 brain concentrations remained low in warfarin treated rats suggesting an alteration of the MK-4 biosynthetic pathway in the presence of warfarin (53). Moreover, rats treated with VKAs showed worse performances in tests to evaluate their cognitive and behavioral functions (53, 54).

It is well known how anticoagulant use can decrease the risk of dementia by reducing the number of cerebral ischemic events in AF patients (55).

Mostaza et al. observed that in a population taking vitamin K antagonists, there was a trend toward higher warfarin prescription among patients with cognitive impairment, regardless dependency or frailty. Thus, a thorough evaluation on the association between non-vitamin K antagonists oral anticoagulants (NOACs) use and cognitive decline is crucial (56).

A recent meta-analysis found a borderline significant association between the use of NOACs and the lower risk of cognitive impairment when compared with VKAs and acetylsalicylic acid (57).

Similar findings were observed in other studies where NOAC were considered an optimal or even better alternative to warfarin, due to their lower bleeding risk and variability in anticoagulation effect (58). A previous study validated the same hypothesis: NOACs provide a better protection against atrial fibrillation-related stroke in terms of lower risk of cerebral ischemic events and new-onset dementia than those treated with warfarin (59).

## Vitamin K and Cognitive Decline

Considering the numerous roles of Vitamin K highlighted in the previous studies, in recent years some authors have started to investigate the potential link between cognitive impairment and vitamin K.

Whether vitamin K deficiency is associated to cognitive decline is still a matter of debate today. From the literature search, we were able to include 7 human studies and all, except one (60), confirmed an association between vitamin K and cognitive function among older adults (Table 1).

**Table 1:** Studies on vitamin K concentration and cognitive performances.

**References**	**Participants**	**Design**	**Vitamin K determination**	**Cognitive evaluation**	**Main results**	**Strenghts**	**Limitations**
Sato et al. (61)	100 demented women (mean age 81), 100 cognitively normal female controls (mean age 81)	Cross Sectional	Standardized HPLC^a^ procedures	MMSE^b^	Patients with severe dementia found to have a lower Vitamin K serum concentration	Reliable methodology for serum levels of vitamin K	No follow-up, relatively small sample size
Presse et al. (62)	31 AD^c^ patients and 31 healthy controls (mean age 78)	Cross Sectional	Vitamin K intake measured using 3- or 4-days diet records	MMSE^b^	9 patients with AD^c^ found to have a lower intake of vitamin K	Accuracy of food records ensured with a dietitian and the help of caregivers	Dietary intake data of limited value for assessing vitamin K status, small sample size, potential over selection of patients
Presse et al. (63)	320 elderly (mean age 76) from Québec Longitudinal NuAge Study	Cross Sectional	Standardized HPLC^a^ procedures	MMMSE^d^, 6 tests covering 4 cognitive domains	Positive association between higher vitamin K intake and verbal episodic memory	Comprehensive cognitive battery assessing 4 cognitive domains, large sample size, reliable methodology for serum levels of vitamin K	No follow-up, potential confounders (such as ApoE genotype) potential over selection of patients
Van der Heuvel et al. (60)	599 participants (mean age 60) from the Longitudinal Aging Study Amsterdam	Prospective (follow up: 6 years)	dp-ucMGP^e^	Alphabet coding task, Auditory verbal learning test, Raven's colored progressive matrices	No association found between dp-ucMPG^e^ and cognitive functions	Inclusion of a range of cognitive tests sensitive to aging measured over 6 years follow-up	Indirect measure of vitamin K status (dp-ucMGP^e^), only a single measurement of Vitamin K status was available
Chouet et al. (64)	192 patients (mean age 83) recruited from the Cognition and LIPophilic vitamins study	Cross Sectional	FFQ^f^	MMSE^b^, FBRS^g^	Positive association between vitamin K intake and MMSE^b^ and FBRS^g^ scores	Assessment of the dietary vitamin K intake over 12 months, assessment of both cognitive and behavioral outcomes	FFQ^g^ validated on Canadian population (tends to overestimate intakes), poor estimation of Vitamin K intake in patients with cognitive disorders
Soutif-Veillon et al. (65)	160 patients (mean age 82) recruited from the Cognition and LIPophilic vitamins study	Cross Sectional	FFQ^f^	MAC-Q^h^	Positive association between vitamin K intake and memory complaint questionnaire score	Assessment of the dietary phylloquinone intake over 12 months	Use of MAC-Q^h^ tool may have overestimated the prevalence of subjective memory complaint, poor estimation of vitamin K dietary intake in patients with cognitive disorders
Kiely et al. (66)	156 elderly (mean age 78) from ELDERMET cohort	Cross Sectional	FFQ^f^ and HPLC^a^	MMSE^b^	Serum and dietary phylloquinone were significant and independent predictors of good cognitive function	Simultaneous measurement of both dietary and serum phylloquinone	Self-selected, motivated cohort, with potentially better diet, education, and lifestyle than general population

a *High Performance Liquid Cromatography*.

b *Mini-Mental State Examination*.

c *Alzheimer's Disease*.

d *Modified Mini-Mental State Examination*.

e *Desphospho-uncarboxylated matrix Gla protein*.

f *Food Frequency Questionnaire*.

g *Frontotemporal Behavioral Rating Scale*.

h *Memory Complaint Questionnaire*.

Six studies demonstrated, in a population of 65 years and older, a direct correlation between low vitamin K dietary intake or serum concentration and deteriorated cognitive and behavioral performances. In particular, Presse et al. in 2013 published the results of a cross sectional study conducted on 320 elderly, aged 70–85, free of cognitive impairment from the NuAge study cohort (63). Phylloquinone serum concentration analysis was measured using High performance liquid chromatography (HPLC), a method already validated as an indicator of dietary phylloquinone intake over a long period of time. Circulating phylloquinone concentrations are conditioned by the blood lipid profile that needs to be assessed as well while using HPLC (67). Cognitive assessment was performed using specific tests for each cognitive domain (verbal and non-verbal episodic memory, executive functions, and speed of processing). Results showed that recruited subjects with higher serum phylloquinone performed better in verbal episodic memory, while no correlation was found with non-verbal episodic memory, executive functions, and speed of processing, underlining the role of vitamin K in memory consolidation (63). These results are supported by a previous murine model where rodents underwent a 5-days learning test in the Morris Water Maze and those fed with lower vitamin K levels required longer time to perform the task compared to those adequately fed (20).

Two studies evaluated vitamin K intake using a semi quantitative food frequency questionnaire (FFQ). They observed a less severe subjective memory complaint (65) as well as a better cognition and less behavioral disturbances (64) among geriatric patients with higher vitamin K intake. The FFQ is a 50 items questionnaire that aims to evaluate vitamin K dietary intake. Although it has been validated in elderly people (68) it may result in an underestimation when investigating patients with cognitive decline. Therefore, the FFQ may not be as representative of phylloquinone intake as its serum levels measured with HPLC in this specific circumstance.

In line with previous studies, Kiely et al. described that elderly with poor cognitive functions, evaluated with MMSE, had the lowest dietary vitamin K intake (assessed with FFQ). Similar results were observed correlating MMSE scores and phylloquinone serum levels measured with HPLC (66).

Further publications highlighted the potential role of this vitamer among patients with Alzheimer's disease-related dementia (61, 62), who were found with significant lower levels of vitamin K even after data were adjusted for energy intakes. In particular, Presse et al. reported in 2008 how vitamin K intake, evaluated with 3–5 days diet records, were notably lower in patients with early stages of AD. Partially limiting the strength of this study are the diet records used, as they show limited value for the assessment of vitamin K intake (62).

Opposite results were obtained by Van Den Heuvel et al. (60) in a middle-age sample (55–65 years) of 599 individuals, measuring vitamin K in an indirect way through levels of desphospho-uncarboxylated matrix Gla-protein (dp-ucMGP). Over the 6 years follow up, no significant association between vitamin K and cognitive decline was found. The recruitment criterion of a different age group may be the reason why contrasting results were observed, since the brain might be susceptible to nutrient deficiency in different ways at different times in life (20). Moreover, as stated by the authors themselves, dp-ucMGP may not be the most suitable marker of vitamin K levels in the brain (60).

An emerging issue is how the use of VKAs could influence brain metabolism and this topic is analyzed separately. The few papers published until now point out, to a limited extent, a potential correlation between the use of VKAs and both cognitive decline and brain focal atrophies (Table 2).

**Table 2:** Studies on VKAs and cognitive performances.

**References**	**Participants**	**Design**	**Cognitive evaluation**	**Main results**	**Strenghts**	**Limitations**
Annweiler et al. (69)	267 patients (mean age 83) hospitalized or seen in consultation from WARHOL^a^ study	Cross Sectional	MMSE^b^	Fluindione is positively associated to lower MMSE^b^ score	Standardized collection of data, detailed description of the participants' characteristics	Restricted study cohort, cognitive impairment assessed using only MMSE^b^ score, no serum vitamin K concentration evaluated, association between cognitive impairment and the use of VKAs^c^ accounted neither for the length of treatment nor for INR^d^ history
Ferland et al. (70)	7,133 nondemented community-dwellers (mean age 73)	Prospective (follow up: 10 years)	IST^e^, BVRT^f^, MMSE^b^	VKAs^c^ usage is positively associated to lower IST^e^ and BVRT^f^ scores, no association was found between vitamin K and MMSE^b^	Large population cohort, follow-up of 10 years	No dietary Vitamin K intake or serum levels evaluated, no detailed information of VKAs^c^ treatment (doses, length), limited number of cognitive tests available at each follow-up
Brangier et al. (71)	18 VKAs^c^ and 36 community-dwellers from GAIT^g^ cohort (mean age 76)	Cross Sectional	None (MRI scans for brain volumery)	The duration of exposure to VKAs^c^ correlated with focal brain atrophy	Use of VBM^h^ approach to locate focal atrophies, standardized collection of data from a single research center	Geriatric study cohort, use of two different MRIs (1,5T and 3T), no information provided by VBM^h^ analysis on the potential mechanisms causing atrophies, segmentation, and normalization defect in VBM^h^ analysis
Brangier et al. (72)	378 geriatric outpatients (46 VKAs^c^ users) from MERE^i^ study (mean age 82)	Prospective (follow up: 24 months)	MMSE^b^, FAB^j^	Use of VKAs^c^ was associated with lower FAB^j^ scores at baseline, and with more significant worsening after 24 months. No significant association between MMSE^b^ scores and VKAs^c^ use.	Longitudinal prospective design with an intermediate mid-term evaluation and a final long-term evaluation, standardized collection of data from a single research center	Geriatric study cohort, incomplete follow-up at 12 and 24 months, no detailed information of VKAs^c^ treatment (length, history of INR^d^), no dietary vitamin K intake or serum levels evaluated

a *Who is At Risk of Hypovitaminosis in Older study*.

b *Mini-Mental State Examination*.

c *Vitamin K Antagonists*.

d *International Normalized Ratio*.

e *Isaac Set Test*.

f *Benton Visual Retention Test*.

g *Gait and Alzheimer Interactions Tracking*.

h *Voxel Based Morphometry*.

i *Alzheimer's Disease and Related Disorders' study*.

j *Frontal Assessment Battery*.

Ferland et al. observed in a large cohort study a significant decrease in visual memory and verbal fluency among patients treated with VKAs when compared to individuals under no blood-thinning treatment, but no association was observed between the global cognitive function and VKAs use over at 10 years follow up (70).

Brangier et al. analyzed the brain volume, using 3 or 1.5 Tesla MRI, of 54 subjects (18 under VKAs treatment and 36 matched controls) and found a significant inverse correlation between the duration of drug exposure and gray/white matter volume in the right frontal inferior operculum, right precuneus, and left middle frontal gyrus (71). The same author found an important decline in executive functions (assessed with frontal assessment battery) among geriatric patients treated with VKAs over at 24 months follow up. It's worth noticing how, in the same study, decline in Mini mental state examination (MMSE) scores, used as an assessment of cognitive performance, was not found significantly associated with VKAs use over the same period of time (72).

Lastly, Annweiler et al. in 2015 reported a 15% higher risk of cognitive impairment in patients treated with fluindione which is used as an anticoagulant drug. The positive association (not observed in patients treated with warfarin or acenocoumarol) remained statistically significant even after adjustment for covariables (69).

Clinical studies have suggested that VKAs use does not affect vitamin K plasma concentration (73) and involved molecular patterns that might lead to cognitive impairment in humans using VKAs are yet to be comprehended.

When implying an involvement of VKAs in cognitive impairment it must be considered that patients are under treatment for pathologies like atrial fibrillation, that can possibly influence, to some level, mental deterioration (74).

## Conclusions

The present review stems from a growing interest in the role of vitamin K in brain function, especially in cognition. It collected recent contributions to the topic, showing interesting, even though not definitive, evidence of direct correlation between vitamin K levels and cognitive performance. Moreover, VKAs might influence negatively some cognitive domains such as visual memory and verbal fluency and the brain volume. Only a small number of publications were based on studies performed on humans, limiting the amount of papers included. These studies were heterogeneous in several ways: study design, markers used to measure vitamin K levels, method used to assess cognitive performance and age of patients included in the studies. Further evidence should be gathered using more standardized methodology to foster comparability of results.

The paucity of published papers suggests the need of a more thorough investigation from the scientific community, using randomized trials with large samples to confirm the hypothesis that low vitamin K can be associated to cognitive decline. A standardized methodology for both cognitive evaluation and vitamin K dietary intake and serum concentrations must be adopted in order to develop more comparable and reliable data.

Due to the large number of individuals treated with VKAs, a large prospective study is possible and could be crucial to elucidate the influence of these drugs on vitamin K serum levels and consequently on cognitive decline.

In conclusion, considering the growing social and economic burden linked to the increasing number of patients suffering from cognitive impairment and dementia, further researches on this topic can prove to be beneficial and applicable results can be expected.

## Author Contributions

MF and LA conceived the original idea. LA, CD, and RC organized the database and wrote the various sections of this review. AC, MF, FC, AL, and GC supervised and made sure that this work was accurate and formally correct. All authors reviewed the manuscript before the submission.

### Conflict of Interest Statement

The authors declare that the research was conducted in the absence of any commercial or financial relationships that could be construed as a potential conflict of interest.
